# Evaluating discussion board engagement in the MoodSwings online self-help program for bipolar disorder: protocol for an observational prospective cohort study

**DOI:** 10.1186/s12888-015-0630-7

**Published:** 2015-10-14

**Authors:** Emma Gliddon, Sue Lauder, Lesley Berk, Victoria Cosgrove, David Grimm, Seetal Dodd, Trisha Suppes, Michael Berk

**Affiliations:** IMPACT Strategic Research Centre, Deakin University, Geelong, Australia; The Department of Psychiatry, The University of Melbourne, Parkville, Australia; The Collaborative Research Network, Federation University, Ballarat, Australia; Orygen - The National Centre of Excellence in Youth Mental Health, Parkville, Australia; School of Psychology, Deakin University, Burwood, Australia; VA Palo Alto Health Care System, Palo Alto, California USA; Stanford University School of Medicine, Stanford, California USA; Florey Institute for Neuroscience and Mental Health, Parkville, Australia

**Keywords:** Bipolar disorder, Internet, Online, Support group, Mental health

## Abstract

**Background:**

Online, self-guided programs exist for a wide range of mental health conditions, including bipolar disorder, and discussion boards are often part of these interventions. The impact engagement with these discussion boards has on the psychosocial well-being of users is largely unknown. More specifically we need to clarify the influence of the type and level of engagement on outcomes.

The primary aim of this exploratory study is to determine if there is a relationship between different types (active, passive or none) and levels (high, mid and low) of discussion board engagement and improvement in outcome measures from baseline to follow up, with a focus on self-reported social support, stigma, quality of life and levels of depression and mania. The secondary aim of this study is to identify any differences in demographic variables among discussion users.

**Methods/design:**

The present study is a sub-study of the MoodSwings 2.0 3-arm randomised controlled trial (discussion board only (arm 1), discussion board plus psychoeducation (arm 2), discussion board, psychoeducation plus cognitive behavioural therapy-based tools (arm 3)). Discussion engagement will be measured via online participant activity monitoring. Assessments include online self-report as well as blinded phone interviews at baseline, 3, 6, 9 and 12 months follow up.

**Discussion:**

The results of this study will help to inform future programs about whether or not discussion boards are a beneficial inclusion in online self-help interventions. It will also help to determine if motivating users to actively engage in online discussion is necessary, and if so, what level of engagement is optimal to produce the most benefit. Future programs may benefit through being able to identify those most likely to poorly engage, based on demographic variables, so motivational strategies can be targeted accordingly.

**Trial registration:**

ClinicalTrials.gov NCT02118623 registered April 15 2014 and NCT02106078 registered May 16 2013.

## Background

The growth of the internet has led to new opportunities for people seeking adjunctive self-guided health programs. Online psychotherapeutic interventions have now been trialled in a wide range of mental illnesses [1], including bipolar disorder [2]. Many of these interventions involve online discussion boards, also known as discussion forums [3], to allow users to communicate with other likeminded peers. It is unclear, however, what impact these boards have on the psychosocial outcomes of users. It is even less clear how users interact with discussion boards (discussion engagement), and how this might influence outcomes. There are different types of engagement, with some users actively engaging by contributing new content, while others passively engage through reading [4]. There are also different levels of this engagement (e.g. low, mid or high), depending on how frequently users engage. To date, there are no published studies analysing the impact of the different types and levels of discussion engagement on psychosocial outcomes in users of discussion boards within online psychotherapeutic interventions. Greater clarity is needed regarding what role discussion boards play within online interventions, what benefits can be gained through discussion board participation, and what level or type of engagement, if any, is required to gain the most benefit from these discussion boards. This paper describes an ongoing study evaluating discussion board engagement and its impact on perceived social support, quality of life, stigma and symptom severity within an online self-help program for bipolar disorder, known as MoodSwings 2.0.

**Table:** 

**Data category**	**Information**
Primary registry and trial identifying number	ClinicalTrials.gov
	NCT02118623
	NCT02106078
Date of registration in primary registry	April 15 2014 (NCT02118623)
	May 16 2013 (NCT02106078)
Secondary identifying numbers	ACTRN12614000409673, U1111-1155-4445, 1R34MH091384, 1R34MH091284
Source(s) of monetary or material support	National Institute of Mental Health
Primary sponsor	University of Melbourne
Secondary sponsor(s)	VA Palo Alto Health Care System
Contact for public queries	Emma Gliddon, BAppSci(Psych), BSci(Hons)
	Deakin University
	+61342153311 eglid@deakin.edu.au
Contact for scientific queries	Prof Michael Berk, FFPsych, PhD
	Deakin University
	MIKEBE@BarwonHealth.org.au
Public title	Internet-Based Interventions for Bipolar Disorder (MoodSwings 2)
Scientific title	Sub-study of a Randomized Trial of Internet-Based Interventions for Bipolar Disorder
Countries of recruitment	Worldwide
Health condition(s) or problem(s) studied	Bipolar disorder
Intervention(s)	Moderated discussion board
Key inclusion and exclusion criteria	Inclusion Criteria:
	- Current diagnosis of bipolar I disorder, bipolar II disorder, or bipolar disorder not otherwise specified (NOS) verified with the Structured Clinical Interview for the Diagnostic Manual for Mental Disorders (SCID) mood disorders module.
	- Age 21 to 65
	- Access to a computer with internet access.
	- Able to speak and read English proficiently.
	- Some degree of medical supervision of bipolar disorder (sees a health professional at least twice a year to discuss symptoms and treatment needs).
	- Local access to emergency care.
	Exclusion Criteria:
	- Current psychosis, as assessed in screening phone interview with the SCID psychotic screening module.
	- Acutely suicidal (defined as having a Hamilton Rating Scale for Depression [HAM-D] item 3 scores of = 3)
	- Current mania, assessed using the SCID mood disorder module.
Study type	Observational prospective cohort design
	Allocation: None
	Intervention model: Single group
	Masking: N/A
Date of first enrolment	February 2014
Target sample size	300
Recruitment status	Recruitment complete
Primary outcome(s)	Medical Outcomes Study Social Support Survey (MOS-SSS) [Time Frame: Change from Baseline to 3 months, 6 months, 9 months and 12 months]
	Inventory of Stigma Experiences [Time Frame: Change from Baseline to 3 months, 6 months, 9 months and 12 months]
	Quality of Life Enjoyment and Satisfaction Questionnaire (Q-LES-Q) [Time Frame: Change from Baseline to 3 months, 6 months, 9 months and 12 months]
	Montgomery-Asberg Depression Rating Scale (MADRS) [Time Frame: Change from Baseline to 3 months, 6 months, 9 months and 12 months]
	Young Mania Rating Scale (YMRS) [Time Frame: Change from Baseline to 3 months, 6 months, 9 months and 12 months]
Key secondary outcomes	

### Potential benefits of discussion engagement

A number of features of online discussion boards set them apart from other online communication mediums such as social networking sites (e.g. Facebook) and chat services. Unlike most other online communication mediums, the use of pseudonyms in online discussion boards is commonplace, which means members cannot personally identify each other. Suler [5] suggested this concealment of one’s true identity acts as “dissociative anonymity”, where Internet users create an online psyche, separate from their day to day self. This contributes to what has been described as the “online disinhibition effect”, where people feel more open and willing to disclose information about themselves they might ordinarily find embarrassing or stigmatizing [5]. Discussion forums also offer asynchronous communication (i.e. users do not have to be online at the same time to communicate), rather than the real-time, instant communication of live chat. This not only gives users the opportunity to take their time in writing out a thought-out response, but also allows time to prepare for others’ responses. Removing the pressure to hear immediate reactions from others boosts the disinhibition effect. Asynchronous discussion also allows users to disclose information and leave without having to deal with responses from others, which can be particularly comforting for those sharing emotional or personal posts who simply wish to “put it out there” rather than receive feedback from others [5].

People seek out online discussion forums for a variety of reasons. Townsend, Gearing and Polyanskaya [6] found a fear of being hospitalised or taking medications, and poor insurance coverage were significant predictors of mental health-related online forum use. These findings suggest people see a greater role for online discussion forums when face-to-face healthcare isn’t accessible or is anxiety provoking. People may also seek out discussion boards to expand their social networks. It has also been suggested that online discussion forums open up a much broader social network of similar peers that one would not otherwise have access to offline [7]. This may be particularly appealing to those suffering from a stigmatized illness, such as bipolar disorder, where people can often feel unable or inhibited to discuss their illness with others who do not have personal experience.

The possible benefits of engagement with online discussion boards include increased social support, a reduced perception of stigma and improved quality of life, which are of central importance to people with bipolar disorder [8].

#### Increased social support

The term social support refers to the availability of interpersonal resources, and more specifically has been defined as any information that leads an individual to believe they are cared for and loved, esteemed and valued, and/or belong to a network of communication and mutual obligation [9]. Social support can be categorised into five key types: informational (providing information or advice), tangible (providing, or offering to provide goods or services), esteem (communicating respect and confidence in abilities), emotional (communicating love, concern or empathy) and social network support (communicating belonging to a group of people with similar experiences) [10, 11].

In bipolar disorder, social support has been identified as a potential protective factor against suicide [12, 13]. A lack of social support has also been identified as a factor influencing the risk of relapse [14], the recurrence of bipolar depression [15], and reduced psychosocial functioning [16]. The opportunity to provide social support online could therefore have a significant impact on those living with bipolar disorder.

A study looking specifically at self-help discussion boards for bipolar disorder found social networking was the major theme of discussion [17], with the largest percentage of postings including statements about social networks. These findings suggest that social relationships are seen as important to those with bipolar disorder who are using online forums, suggesting that this may be a perceived role of such forums. Discussion board usage has been linked to an increase in support network size, as well as support network satisfaction [18]. These findings suggest that those participating in online discussion are seeking to broaden their social networks, and discussion boards are providing a valuable space to foster such networks.

#### Reduced perception of stigma

Stigma has been defined in a number of ways, with a series of complex factors involved [19]. Link and Phelan [19] broadly define stigma as a combination of labelling, stereotyping, separation (or “us” and “them” mentality), loss, and discrimination. These attitudes and behaviours are perpetrated by an individual or group who possess some level of social, economic and/or political power [19], and can include close friends and family members, resulting in the loss of previously valuable sources of support [20].

Many factors contribute to reduced social support, including stigma. People who perceive stigma from others may withdraw from social interaction, rather than seek the support and contact they need [21]. For this reason, the perceived absence of stigma in online communities is a potential attraction, with users indicating it is the most commonly perceived advantage of online support when compared with face-to-face support [18].

Due to the anonymous nature of online discussion, there is great potential for discussion boards to aid in reducing stigma, as anonymity is considered a key influence in reducing the level of stigma experienced by online discussion board users [22]. It’s possible that by disclosing more information and receiving positive, supportive responses, users begin to perceive less stigma. Like social support, stigma has been linked to poorer psychosocial functioning in people with bipolar disorder [16]. Disappointingly, the experience of stigma has been self-reported in as much as 85 % of people with bipolar disorder [23].

In face-to-face support groups for mental illness, identification with a group has been shown to significantly increase resistance to the adverse effects of stigma [24]. Group identification is the acknowledgement of belonging to a particular group. It is suggested that this identification provides a basis for social support, which is also significantly associated with resistance against stigma [24]. These positive effects evident in face-to-face groups may be replicated in online discussion boards as they also allow members to identify with similar others. Many people with mood disorders avoid face-to-face support groups completely, due to social anxiety or fear of discrimination [25], and online discussion boards provide an alternative which may help to alleviate these issues.

#### Improved quality of life

Broadly, quality of life (QoL) is defined as an individual’s well-being in a spectrum of life domains, such as occupational, emotional, social and physical functioning [8]. It is unclear whether or not discussion board use helps or hinders quality of life, despite it being positively linked to both social support and stigma.

Along with social support and stigma, QoL is an important factor to consider in illness management for bipolar disorder. It has been identified as a significant area of impairment for those with bipolar disorder and is often regarded as a key target for treatment [8]. A number of online interventions for mood disorders that include discussion boards have shown significant improvements in QoL [2, 26, 27], though only one of these studies specifically evaluated the impact of discussion board use on QoL [27]. Crisp et. al [27] found that when the e-couch online intervention for depression was coupled with an online discussion board, European Health Interview Survey-Quality of Life(EUROHIS-QoL) 8-item index scores significantly improved when compared with the intervention and discussion board separately. These results highlight the potential benefit of including online support groups within online interventions for mental illness, and mood disorders in particular. This study, however, did not evaluate users’ level or type of discussion board engagement, so it remains unclear whether or not particular engagement patterns result in greater benefits to users.

#### The role of discussion engagement

Despite the potential benefits, discussion board utilization can often be limited, with only around 50 % of users posting at least once, and a small minority of users posting the majority of content [26]. Little is known about the percentage of users who read content shared by others (known as lurkers [28]), or what benefit reading activity may have. Most studies into discussion board engagement focus on users who actively contribute new content (known as posters [28]), and many do not take reading behaviour into account at all. Further investigation is required to evaluate the role of discussion board engagement, to determine if active or passive discussion engagement is most influential (if at all), and to determine what level of engagement is required to receive the most benefit.

Despite the growing number of online self-help programs for mental illness, many of which include online discussion boards, no published studies have evaluated discussion board engagement within an online intervention. The current study utilizes the MoodSwings online self-help program for bipolar disorder, which includes a series of educational learning modules, interactive tools, and three moderated peer discussion boards. This program offers a valuable opportunity to: Observe differing levels of active and passive discussion engagement in comparison to those who do not engage at all; evaluate the impact of discussion board engagement on both self report and clinician administered psychosocial outcome measures; and view discussion engagement in the context of an online self-help intervention.

## Aims

The primary aim of this novel exploratory study is to determine if there is a relationship between discussion board engagement and improvement in outcome measures from baseline to follow up, with a focus on self-reported social support, stigma, quality of life and levels of depression and mania. The secondary aim is to identify any differences in demographic variables between users who have different types and levels of discussion board engagement (active, passive or none).

## Methods/design

The present study is a sub-study of a National Institute of Mental Health (NIMH) R34 funded randomised controlled trial evaluating the efficacy of the MoodSwings online self-help program for bipolar disorder. This sub-study involves an analytic observational prospective cohort design using a naturalistic sample. This sub-study focuses on the three discussion boards included within the MoodSwings program. While there was no allocation to specific conditions by the researchers for this sub-study, it does take place within the context of a RCT that involves randomly allocating participants to three graded but parallel groups, however all participants receive a discussion board. Individuals have access to one of three different treatment arms of the MoodSwings program, and the discussion boards are allocated per treatment arm. Arm one has access to a discussion board alone; arm two has access to a discussion board and a series of online learning modules; and arm three has access to a discussion board, the learning modules, and a number of interactive tools. In this sub-study, the researchers observed and categorised participants’ behaviour and examined effects on outcomes prospectively.

### Ethics, consent and permissions

This study was given ethical approval by the Barwon Health Human Research Ethics Committee (Project ID 11/73) and the Institutional Review Board for Stanford University (Project ID 21897). Informed consent was collected from all participants via online and/or mailed forms prior to eligibility screening. All data collected is entered into a secure, password protected electronic database in a de-identified format.

### Participants

A total of 304 participants have been recruited worldwide, with research teams based in Geelong, Australia and Palo Alto, United States. The majority of participants were recruited via social media and other online advertisements. To be eligible for entry into the MoodSwings program, participants met inclusion and exclusion criteria (see Table 1). The MoodSwings program is an adjunctive self-help program intended to be utilized in additional to usual care, so participants were not prohibited from partaking in other interventions and/or treatment during the study period. Individual participation in this study may be discontinued at any time at the request of a participant.

**Table 1:** Inclusion and exclusion criteria for the MoodSwings program

Inclusion criteria	Exclusion criteria
Aged 21 to 65 years	Current psychosis
Diagnosis of bipolar disorder	Current mania
Access to a computer with internet	Acutely suicidal
Able to speak and read English proficiently	
Current health professional supervision for bipolar disorder	
Local access to emergency care	

### Procedures

All participants have access to the MoodSwings program and discussion boards for 12 months. Over this study period, participants are able to submit posts to one of the three discussion boards, allocated per treatment arm. The discussion boards are moderated by research staff who are supervised by a clinician. All posts submitted to each discussion board are screened by a moderator before being published for other participants to see and all posts are moderated within two business days of submission. To be approved, all posts must fall within a set of guidelines which outline appropriate content. Inappropriate content including personal information, offensive language and distressing content is not allowed. Posts indicating suicidal ideation or intention will be followed up via phone or internal message within one business day of moderation. Posts discussing previous suicidal experiences are carefully screened and only approved if deemed to be beneficial to the group. Topics containing such posts are labelled with a trigger warning.

Moderators post one discussion topic per month across all three discussion boards for the duration of the study. This is intended to generate discussion and ensure all users have access to new discussion material regardless of when they enter the program, as users will be commencing the study at different times across the recruitment period. Discussion topics are broad but relevant to people with bipolar disorder, with some topics generated based on discussion themes identified in a previous trial of the MoodSwings program [2], and some relating to the topics covered in the learning modules of the MoodSwings program. Topics are determined on an ongoing basis throughout the study to ensure moderator topics do not duplicate existing topics created by users.

### Measures

#### Eligibility

All participants were screened via phone prior to their commencement in the MoodSwings program to confirm their eligibility. A current diagnosis of bipolar disorder was confirmed via the Structured Clinical Interview for the DSM-5 [29], and other screening measures were used to determine the remaining criteria (see Table 2).

**Table 2:** Schedule of assessments

	Screening	Baseline	3	6	9	12
Phone assessment						
Structured Clinical Interview for DSM-5 (Research Version) (SCID-5-RV) [29]						
- Mood modules	X					
- Psychotic screen module	X					
Hamilton Depression Rating Scale item 3 (HAM-D) [32]	X					
Young Mania Rating Scale (YMRS) [31]		X	X	X	X	X
Montgomery-Asberg Depression Rating Scale (MADRS) [30]		X	X	X	X	X
Self-Report Online Assessment						
Demographics		X				
Social Support Survey (MOS-SSS) [33]		X	X	X	X	X
Inventory of Stigmatizing Experiences [34]		X	X	X	X	X
Quality of Life Enjoyment and Satisfaction Questionnaire (Q-LES-Q) [35]		X	X	X	X	X

#### Discussion board engagement

Data collected includes post title, post content, time of post, date of post, username, and allocated treatment arm number. All posts submitted to each discussion board are stored for analysis, including those deemed inappropriate for publication. In addition to text content, all participant discussion usage will be tracked to determine discussion engagement patterns. The MoodSwings website allows administrators to monitor page views and log in times, and therefore monitor adherence to the intervention. Discussion board page views will be used to identify passive and active forum activities (i.e. reading and posting activities). These activities will be added up to form active and passive engagement scores, which will then be combined to form a third total engagement score. Tertiles will be calculated for each score to determine cut-off points for low, mid and high level engagement. Each score will then be categorised accordingly, and all three categorised scores will be used to represent each user’s discussion engagement level.

#### Quarterly assessment

Participants who were deemed eligible were then assessed via phone for baseline symptom scores using the Montgomery-Asberg Depression Rating Scale (MADRS) [30] and the Young Mania Rating Scale (YMRS) [31]. These assessments are repeated at 3, 6, 9 and 12 months follow up. Phone assessments will be attempted regardless of participant adherence. Participants are also required to complete a series on online self-report assessments at baseline and 3, 6, 9 and 12 months follow up. These online assessments that are included in this sub-study are shown in Table 2.

### Structured clinical interview for DSM-5 (research version) (SCID-5-RV)

Confirmation of current bipolar disorder diagnoses is gathered via phone using the mood modules from the *Structured Clinical Interview for DSM-5 Axis I Disorders* (Research Version)[29]. This measure is also used to exclude those with current mania, and the psychotic screen module is used to exclude current psychosis.

### Hamilton depression rating scale (HAM-D)

This is a 21-item scale used to assess symptoms of depression. Item 3 of this measure is used in this study to exclude those who are acutely suicidal (HAM-D item 3 score of ≥3) [32].

### Young mania rating scale (YMRS)

This is an 11-item clinician administered scale used to assess symptoms of mania. Previous evaluations of this scale found it to be both a reliable and valid measure of manic symptoms, which is also sensitive to changes over time [31].

### Montgomery-Asberg depression rating scale (MADRS)

This is a 10-item clinician administered scale used to assess symptoms of depression [30]. It is a widely used, valid and reliable measure of depression symptoms, which is particularly sensitive to changes over time [30].

### Demographics

Basic demographic information is collected (e.g. age, gender, employment status etc.), as well as more specific information on illness and treatment history. A single item summarizing participant use of the internet is also included to assess comfort and familiarity with internet usage.

### Medical outcomes study – social support survey (MOS-SSS)

This 18-item self-report scale was developed to assess the level of social support across 4 sub-domains (emotional/informational support, tangible support, affectionate support, and positive social interaction) in those with chronic health conditions. It is both a reliable (alpha >0.91) and valid measure of social support [33].

### Inventory of stigmatizing experiences

This self-report questionnaire consists of two subscales: The 10-item Stigma Experiences Scale (measuring the scope of experienced stigma) and the 7-item Stigma Impact Scale (measuring the psychosocial impact of stigma) [34]. A previous evaluation found both subscale to be reliable [34].

### Quality of life enjoyment and satisfaction questionnaire (Q-LES-Q)

This 16-item self-report measure is designed to assess satisfaction with a number of areas of daily life (relationships, living environment, leisure activities, health, medical treatment, and overall satisfaction) [35]. A previous evaluation found the scale is both a valid and reliable measure of quality of life [36].

#### Statistical methods

Quantitative analyses will utilized to identify changes in psychosocial outcomes relating to both active and passive discussion engagement, as well as demographic differences between active, passive and non-user types.

A sample size calculation was conducted prior to the commencement of the study, which determined a total sample of 300 participants would be required to reach 80 % power with a significance level of 0.05, across 4 assessment points, taking 20 % attrition into consideration.

#### Study monitoring

##### Data monitoring

A Data Safety and Monitoring Board (DSMB) has been established for the MoodSwings randomised controlled trial, and therefore this sub-study. The DSMB is comprised of three psychiatrists based in the United States and a senior pharmacist based in Australia, all of whom are independent of the study. The DSMB’s role in this study is to review procedures; discuss scientific and ethical issues; monitor protocol compliance; and monitor data collection, management and quality. The DSMB has a reporting responsibility to the funding body and each research sites’ local governance body (Institutional Review Board or Human Research Ethics Committee).

##### Participant safety

Information regarding adverse events is collected during quarterly phone assessments (3, 6, 9 and 12 months follow up). Participants also have the opportunity to report events to research staff at any time via online private message. Adverse events are reported to the principal investigators and DSMB within 24 hours of research staff becoming aware.

## Discussion

Online discussion boards have been shown to have great potential in improving social support, reducing perceived stigma [18] and improving quality of life [27]; however it is unclear what type or level of engagement is necessary to reap any benefit and what structures facilitate or inhibit such engagement. The MoodSwings online psychotherapeutic intervention for bipolar disorder offers a valuable opportunity to deliver online discussion boards in an environment where natural usage patterns can be observed and accurately monitored. This will allow clear comparisons between users and non-users, as well as users with differing types and levels of engagement on a broad range of both self-report and clinician administered outcome measures. The results of this study will help to inform future programs about whether or not discussion boards are a beneficial inclusion in online self-help interventions. It will also help to determine if motivating users to actively engage in online discussion is necessary, and if so, what level of engagement is optimal to produce the most benefit. Future programs may benefit through being able to identify those most likely to poorly engage, based on demographic variables, so motivational strategies can be targeted accordingly.

As the Internet grows, there is increased appreciation of the utility of online discussion boards, particularly in the area of mental health. This research will assist in clarifying and maximising the benefits of online discussion, provide data guiding the design and utilisation of discussion boards, and will give clinicians and online users the best chance of positively utilizing online mental health communities.

## Abbreviations

EUROHIS QoL: European Health Interview Survey-Quality of Life
HAM-D: Hamilton Depression Rating Scale
MADRS: Montgomery-Asberg Depression Rating Scale
MOS-SSS: Medical Outcomes Study – Social Support Survey
NIMH: National Institutes of Mental Health
Q-LES-Q: Quality of Life Enjoyment and Satisfaction Questionnaire
QoL: Quality of Life
SCID-5-RV: Structured Clinical Interview for DSM-5 (Research Version)
VA: Veterans’ Affairs
YMRS: Young Mania Rating Scale
